# *MYO*, a Candidate Gene for Haploid Induction in Maize Causes Male Sterility

**DOI:** 10.3390/plants9060773

**Published:** 2020-06-19

**Authors:** Kimberly Vanous, Thomas Lübberstedt, Rania Ibrahim, Ursula K. Frei

**Affiliations:** Department of Agronomy, Iowa State University, Ames, IA 50011-1051, USA; kbrown@iastate.edu (K.V.); thomasl@iastate.edu (T.L.); rania_1981_akeel@yahoo.com (R.I.)

**Keywords:** haploid induction, maize, male sterility, myosin, RNAi, transgenic, *Zea mays*

## Abstract

Doubled haploid technology is highly successful in maize breeding programs and is contingent on the ability of maize inducers to efficiently produce haploids. Knowledge of the genes involved in haploid induction is important for not only developing better maize inducers, but also to create inducers in other crops. The main quantitative trait loci involved in maize haploid induction are *qhir1* and *qhir8*. The gene underlying *qhir1* has been discovered and validated by independent research groups. Prior to initiation of this study, the gene associated with *qhir8* had yet to be recognized. Therefore, this research focused on characterizing positional candidate genes underlying *qhir8*. Pursuing this goal, a strong candidate for *qhir8*, *GRMZM2G435294* (*MYO*), was silenced by RNAi. Analysis of crosses with these heterozygous RNAi-transgenic lines for haploid induction rate revealed that the silencing of *MYO* significantly enhanced haploid induction rate by an average of 0.6% in the presence of *qhir1*. Recently, *GRMZM2G465053* (*ZmDMP*) was identified by map-based gene isolation and shown to be responsible for *qhir8*. While our results suggest that *MYO* may contribute to haploid induction rate, results were inconsistent and only showing minor increases in haploid induction rate compared to *ZmDMP*. Instead, reciprocal crosses clearly revealed that the silencing of *MYO* causes male sterility.

## 1. Introduction

Doubled haploid (DH) lines are created when cells of haploid seedlings undergo genome doubling, resulting in completely homozygous diploid offspring after self-pollination. DH technology serves as a shortcut for inbred line development, as this procedure only takes two generations. Most haploids utilized in maize breeding programs are created by in vivo maternal haploid induction, which involves crossing a haploid inducer genotype as male to donor genotypes. Current inducers induce haploids at rates of >8% [1,2].

Genetic mechanisms controlling haploid induction rate (HIR) in maize are not well understood. Quantitative trait locus (QTL) mapping has located a major QTL on chromosome 1 [3,4]. The first comparative genome-wide QTL analysis involving inducers UH400 and CAUHOI confirmed that a major QTL, *qhir1*, is present on chromosome 1, and a minor QTL, *qhir8*, was found on chromosome 9 [1]. This minor QTL accounts for up to 20% of the genetic variation and acts to enhance HIR in the presence of *qhir1*. Unlike *qhir8*, *qhir1* is required for haploid induction and accounts for up to 66% of the genotypic variance of this trait [1,5,6]. Dong et al. [7] conducted a fine mapping experiment of *qhir1* using populations from crosses between UH400 and 1680, similar to those of Prigge et al. [1], and were able to narrow the QTL to a 243 kb region. Similarly, fine mapping experiments of *qhir8* using crosses between UH400 and CAUHOI as a mapping population have narrowed *qhir8* to a 789 kb region [5]. Additional regions on chromosome 1 have been detected by Hu et al. [6] with a novel genome wide association study approach detecting selective sweeps. This recently discovered region, *qhir12*, is 3.97 Mb long and is 985 kb downstream of the previously fine-mapped region for *qhir1* [6,7].

In 2017, different research groups independently identified the gene *MATRILINEAL* (*MTL*), which is underlying *qhir1*, and explains the high HIR within inducer lines [8,9,10]. Fine-mapping identified *MTL*, a pollen-specific phospholipase, which was shown to contain a frame-shift mutation in inducer alleles. Furthermore, knockdown lines using RNA interference (RNAi) and complete knockout lines using transcription activator-like effector nuclease (TALEN) for *MTL* were created, resulting in HIRs of 2.7% and 6.7%, respectively.

Prior to the initiation of this study, the gene responsible for *qhir8* had not been identified. However very recently, the gene has been discovered by Zhong et al. [11] who narrowed the fine-mapping region from 789 kb to 138 kb. Within this region, the gene *GRMZM2G465053* (*ZmDMP*) was a strong candidate and expressed during late maturity of pollen grains. By knocking out this gene, HIR was increased 5–6-fold in the presence of qhir1, thereby confirming its likelihood to be responsible for *qhir8*. Additionally, the study shows a significant increase (10–40%) in aborted kernels when this gene is knocked out.

For the current study initiated prior to 2019, candidate genes for *qhir8* were chosen from the study of Kelliher et al. [8], who also conducted RNA-seq experiments between inducer and non-inducer genotypes, resulting in a total of 60 differentially expressed genes, out of which 15 were co-expressed with *MTL* in inducer pollen. One of these genes is *GRMZM2G435294* (*MYO*), located within the 789 kb *qhir8* fine mapping region [5]. Therefore, *MYO* was considered a strong candidate that may be responsible for the enhancement of HIR, when both *qhir1* and *qhir8* are present.

*MYO* is predicted to be a myosin-11 like protein due to several conserved myosin domains, including the myosin N-terminal SH3-like domain (amino acids 10–48), motor head domain (76–720), gap junction protein N-terminal region, and the cargo binding tail domain (1110–1495) [12]. In plants, myosins act to mobilize various vesicular cargoes via attachment to the cargo binding tail domain [13]. *MYO* is likely involved with pollen tube growth, so the function of the cargo binding tail domain is to bind and carry the twin sperm cells. The pollen tube growth is driven by the head motor domain, which binds actin and hydrolyzes ATP, until it fuses with the plasma membrane and allows for pollen tube enlargement and cytoplasmic streaming [14]. Therefore, we predicted the knockdown of *MYO* would have an impact on male fertility. A complete knockout may thus result in male sterility. Instead, reducing *MYO* functions may allow both male fertility and haploid induction. For these reasons, RNA interference (RNAi) is a reasonable option for testing the influence of *MYO* on haploid induction rate.

Herein, we report results from transgenic experiments with altered *MYO* expression in defined genetic backgrounds with regard to presence or absence of *qhir1*. The specific objectives of this research were to (1) explore sequence differences within *MYO* between inducer and non-inducer genotypes, (2) develop transgenic events that successfully silence *MYO* by RNAi, and (3) evaluate these events for HIR and other reproductive phenotypes, such as male fertility.

## 2. Results

### 2.1. Sequence Alignment

*MYO* was confirmed as primary gene of interest because sequencing results revealed several polymorphic regions between inducer genotype RWS, and a non-inducer genotype, B73 RefGen_v3 [15]. There were five different single nucleotide polymorphisms (SNPs) scattered within exon 21. Four out of five SNPs were at the third degenerate position of the codon sequence, all leading to silent mutations. A small insertion of three nucleotides (AGG) was inserted into the RWS sequence at the beginning of exon 21, causing addition of a Glycine. Additionally, a large 60 bp deletion within exon 22 and 23 causes a deletion of 20 amino acids. These changes, however, are all within frame. A deletion of 5 bp within exon 23 causes a frameshift, resulting in 33 amino acid substitutions between B73 and RWS. Following these amino acid changes, there is an introduction of an early stop codon (TAA). The protein length is 1529 amino acids for B73, but with the introduction of the frameshift mutation and the subsequent early stop codon, the protein length is only 915 amino acids within RWS (Table S1), assuming the first exons are intact.

### 2.2. Silencing Efficiency

**Figure 1:**
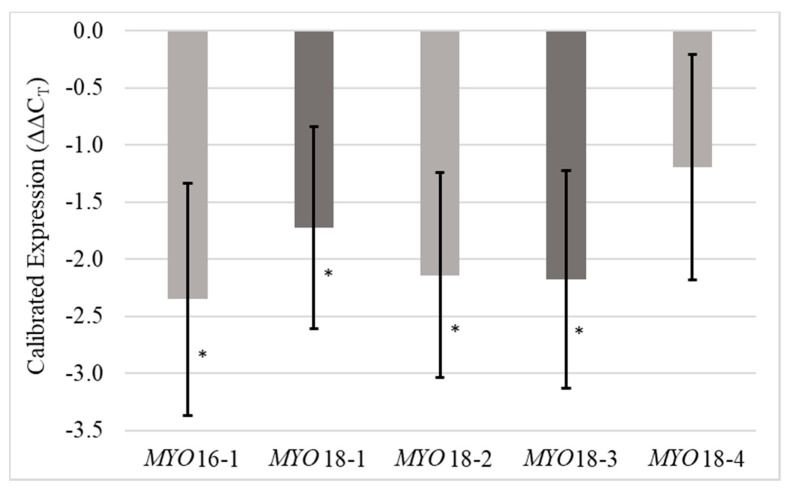
Transgenic mRNA expression normalized to the expression of the house-keeping gene *MEP* and calibrated to the expression of these genes within the wild type (Viking). Error bars represent standard errors. Stars represent significance at the 0.05 level.

Two RNAi constructs (*MYO*16 and *MYO*18) were transformed into a non-inducing genetic background to knock down *MYO*. Five transgenic events, one from *MYO*16 and four from *MYO*18, were backcrossed as BC_1_F_1_ (*^MYO^*BC_1_F_1_) families with B73 and analyzed for silencing efficiency by quantitative PCR (qPCR). Anthers were dissected from immature tassels for RNA extraction. All transgenic events exhibited significantly (*p* = 0.05) decreased expression of *MYO*, and all were significantly different when compared to Viking, except for *MYO*18-4 (*p* = 0.16) (Table 1). Although only one transgenic family from *MYO*16 was included in this study, this construct was most efficient in silencing *MYO*. *MYO*16-1 showed values of −2.35 and 5.09 for calibrated expression levels (ΔΔC_T_) and relative expression levels (fold change), respectively. The average values for *MYO*18 events were −1.81 and 3.63 for calibrated expression levels (ΔΔC_T_) and relative expression levels (fold change), respectively (Figure 1). *MYO*18-4 was removed from the remaining analyses because it was the only transgenic event that did not successfully silence *MYO*.

### 2.3. Haploid Induction Rate

Since *qhir1* is required for haploid induction [1,5,6], it was necessary to evaluate the effect of the transgene (*MYO*) in the presence of *qhir1*. Isogenic lines, with or without *MYO* (RNAi transgene), were created within a *qhir1*-containing B73 genetic background and are denoted as *^qhir1,MYO^*BC_2_F_1_ and *^qhir1^*BC_2_F_1_, respectively. As controls, lines with similar B73 genetic background were created that contained: (1) *MYO*, denoted as *^MYO^*BC_1_F_1_, (2) *qhir1* and *qhir8*, *^qhir1,qhir8^*B73, and (3) *qhir8*, *^qhir8^*B73.

Consistent with expectations [1,5,6], *qhir1* was necessary to achieve higher values of HIR. Three out of the four *^MYO^*BC_1_F_1_ families were not significantly different from the spontaneous HIR of 0.1% (*p*-values ranged 0.03 to 0.19; Table 2). Pooled data from *^MYO^*BC_1_F_1_ families, however, did show significantly different HIR compared to spontaneous rates which was likely resulting from a larger data set and lower error rates (*p* = 0.0051). Pooled HIR data from *^qhir1^*BC_2_F_1_ and *^qhir,1MYO^*BC_2_F_1_ were both significantly higher than spontaneous rates as well, both with *p*-values of <0.0001. Both controls, *^qhir8^*B73 and *^qhir1,qhir8^*B73, also had significantly higher HIR than spontaneous rates with *p*-values of 0.0007 and 0.0016, respectively. Although significantly different compared to spontaneous rates, *^qhir8^*B73 HIR rates were still very low. *^qhir8^*B73, having an HIR of 0.24% (±0.07%), produced lower HIR than *^qhir1,qhir8^*B73, at 1.16% (±0.17%), and was most comparable to *^MYO^*BC_1_F_1_ events, which averaged 0.17% for those that were successfully silencing *MYO*. When comparing *^MYO^*BC_1_F_1_ events to *^qhir8^*B73, only one family showed significant differences with *p*-values of 0.03, 0.17, 0.10, and 0.19 for *MYO*16-1, *MYO*18-1, *MYO*18-2, and *MYO*18-3, respectively. *^qhir1,MYO^*BC_2_F_1_ families scored the highest HIR, averaging 1.53% for those that were successfully silencing *MYO*. In comparison, the isogenic *^qhir1^*BC_2_F_1_ families within these events typically scored lower HIR and averaged 0.96%. When contrasting HIR results from the pooled data of all isogenic families, *^qhir1,MYO^*BC_2_F_1_ was significantly higher than *^qhir1^*BC_2_F_1_ (*p* = 0.02). On average, the addition of *MYO* transgene in a *qhir1* containing background increased HIR values by 0.6%. In addition, the pooled data of *^qhir1,MYO^*BC_2_F_1_ families were not significantly different than *^qhir1,qhir8^*B73 (*p* = 0.27). None of the families, however, displayed significant increases in HIR when comparing *^qhir1,MYO^*BC_2_F_1_ to *^qhir1^*BC_2_F_1_ when analyzed individually. Contrast statements for individual families resulted in *p*-values of 0.73, 0.14, 0.19, and 0.17 for *MYO*16-1, *MYO*18-1, *MYO*18-2, and *MYO*18-3, respectively.

### 2.4. Male Fertility

**Figure 2:**
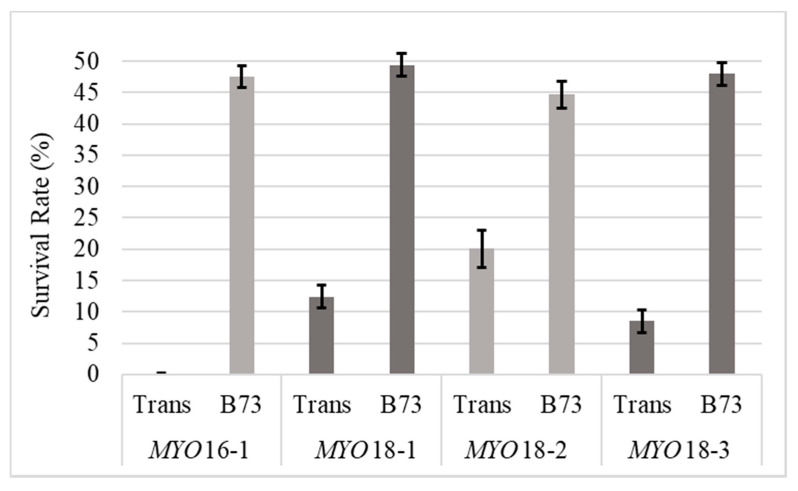
Survival rate of offspring when either *^MYO^*F_1_ (Trans) or B73 were the male parent. All transgenic events were hemizygous for the transgene; therefore, the expected carry over would be 50%. Error bars represent standard errors.

Significant differences were found between events, between differing male parents, and for event and male parent interactions with *p*-values of 0.0001, <0.0001, and 0.0001, respectively (Table 3). There were no significant differences found between events when B73 was the male parent (*p* = 0.4412). Therefore, survival rates were averaged between events and resulted in 47.45% (±1.16%) of offspring carrying the transgene and surviving the herbicide treatment, which was not significantly different than the expected 1:1 segregation ratio of the transgene (*p*-values ranging from 0.27 to 0.96). When *^MYO^*BC_1_F_1_ was the male parent, the portion of offspring carrying the transgene significantly varied between events (*p* < 0.0001) and ranged from 0% (±1.77%) to 20.11% (±2.97%) (Table 3, Figure 2. Interestingly, the transgenic event with the most efficient silencing effect (*MYO*16-1) also resulted in the fewest transgenic offspring when *^MYO^*BC_1_F_1_ was the male parent at 0% (±1.77%). With increasing silencing of *MYO*, there was a corresponding increase in HIR, but also an adverse effect of decreased male fertility (Figure S1).

## 3. Discussion

*MYO* is a strong candidate for the gene underlying *qhir8*, increasing HIR, because it is expressed specifically in anthers and was shown to be up-regulated in wild-type pollen with *MATRILINEAL* (*MTL*), a gene shown to substantially impact HIR and located in *qhir1* [8]. Sequencing of the inducer allele of *MYO* revealed various polymorphic regions affecting the resulting protein. Other polymorphic regions may exist upstream of the gene region sequenced for this study. The frameshift mutation identified in the RWS allele would result in an early stop codon, if the upstream portion of this gene is intact. In addition, a large deletion of 20 amino acids within exons 22 and 23 was detected in RWS relative to B73. This mutation would likely be disruptive to the ability of *MYO* to bind and carry the twin sperm cells, because the truncated protein would be missing the entire cargo binding domain. However, because the motor domain is still present, the elongation of the pollen tube may still happen normally to deliver an “empty” cargo load.

Analysis of HIR among *MYO*-silencing RNAi transgenic families resulted in induction rates of at most 2.0%, which is a very low induction rate considering HIR for inducers exceed 8% [2]. These results were expected, however, because selection with each successive backcross event was focused on the presence of two QTL regions (*qhir1*, *qhir8*) and not for increased HIR, which involves additional QTL. Specifically, with each backcross, offspring were selected for *qhir1* and/or *qhir8* presence by molecular markers. Similar results were obtained by Gilles et al. [9], where *MTL* from inducer genotype PK6 was introgressed over four generations into a non-inducing line, and induction rates fell from 3.59% for BC_0_S_1_ to 0.5% for BC_3_S_1_. These inevitable consequences, due to selection on specific QTL regions, likely result from the absence of unknown QTL regions [1]. In addition, the QTL regions *qhir1* and *qhir8*, as well as the transgene *MYO* were evaluated in the heterozygous or hemizygous state, which was necessary to ensure comparability of segregation patterns between groups. Pollen with induction ability was, therefore, diluted by one half. If two unlinked genetic components were involved, then pollen containing both was diluted even further to one quarter. Considering the comparison of most interest, *^qhir1,MYO^*BC_2_F_1_ vs. *^qhir1^*BC_2_F_1_, only half of the pollen produced from either possesses induction ability mediated by *qhir1*. In *^qhir1,MYO^*BC_2_F_1_, one quarter of the pollen will contain *MYO* only (theoretically having no effect), another quarter will contain *qhir1* only (contributing partially to the HIR), and finally another quarter will have both *MYO* and *qhir1* (*MYO* enhancing haploid induction of *qhir1*).

Results from HIR analysis show that there are two lines of evidence suggesting that *MYO* may be responsible for or contribute to *qhir8*: (1) the majority of *^MYO^*BC_1_F_1_ families were not significantly different from *^qhir8^*B73 while similarly *^qhir1,MYO^*BC_2_F_1_ were not significantly different than *^qhir1,qhir8^*B73, and (2) *^qhir1,MYO^*BC_2_F_1_ had significantly higher HIR than *^qhir1^*BC_2_F_1_. Although most *^MYO^*BC_1_F_1_ families had no significant differences in HIR when compared to spontaneous rates, it is well known that *qhir1* is considered to be required by inducers [1,5,6]. Our results support these earlier findings that *qhir8* depends on the presence of *qhir1 to* increase HIR. Thus, the response from *^qhir1,MYO^*BC_2_F_1_ compared to *^qhir1^*BC_2_F_1_ was of most interest. Although none of the transgenic families showed significant increases from comparing *^qhir1,MYO^*BC_2_F_1_ to *^qhir1^*BC_2_F_1_ when analyzed individually, pooled data resulted in significant differences. In addition, these families, including *MYO*16-1, *MYO*18-1, *MYO*18-2, and *MYO*18-3 exhibited similar or enhanced HIR with the addition of the RNAi construct in the *qhir1* genetic background. This suggests that mutations of this gene in the inducer genotype may be responsible for enhancing HIR by *qhir8* in the presence of *qhir1*.

However, recent findings by Zhong et al. [11] uncovered similar results for gene *GRMZM2G465053* (*ZmDMP*). *ZmDMP* was initially located by narrowing the fine-mapping region of *qhir8* from a 789 kb region [5] to a 138 kb region. *ZmDMP* falls within the narrowed region, however, *MYO* is located approximately 8.5 kb from the boundaries of this region. Zhong et al. [11] found that a knock-out of this gene resulted in a 5–6-fold increase in haploid induction in the presence of *qhir1*. Additionally, they found that when pollinated by the homozygous knock-out of *zmdmp* there was an increase in endosperm aborted kernels of 10–40%. However, there were no significant differences in pollen viability.

By silencing *MYO*, male fertility was affected, as shown by the significantly different percentages of transgenic offspring resulting from reciprocal crosses between transgenic *^MYO^*F_1_ and B73. All families exhibited a substantial and significant decrease in male fertility when *^MYO^*F_1_ was the male parent. The most affected family was *MYO*16-1, resulting in 0% transfer of the transgene when *^MYO^*F_1_ was the male parent, which complements the finding that *MYO*16-1 was also most effective in silencing *MYO*. When B73 is the male parent, the transgene was consistently transferred to approximately half of the offspring.

Considering the expected functions in pollen tube growth of the gene *MYO*, it is not surprising to see some impairment of fertility. It is therefore conceivable that the gene is not completely knocked out in the inducer genome. A complete knockout would likely arrest pollen tube growth, and therefore would result in a male sterile plant. It would be very unlikely that any seed would be produced when pollen tube growth is arrested.

**Figure 3:**
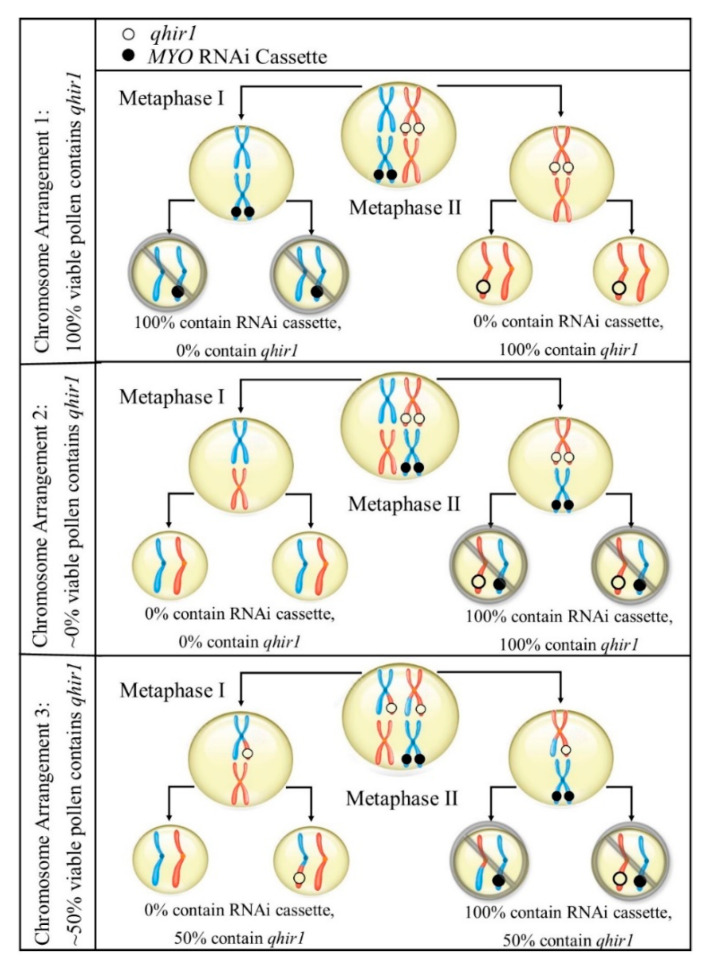
Representation of some potential chromosome arrangements during microsporocyte meiosis within *^qhir1,MYO^*BC_2_F_1_ families. As heterozygotes, crossing over and independent assortment would affect arrangements of both *qhir1* and the RNAi cassette (silencing *MYO*). The dispersal of *MYO* RNAi cassette may produce variable amounts of viable pollen containing *qhir1* (assuming the RNAi cassette causes sterility).

To produce viable seed, including haploids, the pollen tube must reach the ovary to at least fertilize the central nucleate cell to create the endosperm. In that case, if *MYO* is involved with haploid induction, it seems plausible that the mutations of *MYO* within inducer genotype causes only impaired function of the protein. Together with the recent findings from Zhong et al. [11], it seems plausible that the silencing of *MYO* increased haploid induction by chance. For instance, since *^qhir1,MYO^*BC_2_F_1_ and *^qhir1^*BC_2_F_1_ were all maintained as heterozygotes, both *qhir1* and the silencing of *MYO* would theoretically equally affect half of the pollen. However, for *^qhir1,MYO^*BC_2_F_1_, the *MYO* RNAi cassette would cause variable amounts of pollen containing *qhir1* (assuming the RNAi cassette was inserted into a chromosome other than the *qhir1*-containg chromosome). Due to both crossing over and independent assortment, pollen containing *qhir1* may range anywhere from 0% to 100% (Figure 3), which could explain our results. Alternatively, *qhir8* may be comprised of two closely linked genes, both contributing to HIR. *MYO* and *ZmDMP* are closely linked, considering there is only 8.5 kb separating both genes. If both ZmDMP and MYO affect HIR, it would increase the chance for this genome region to be detected as QTL and could explain its consistent detection as major QTL. Similarly, an additional region (*qhir12*) within *qhir1* on chromosome 1 is located 985 kb upstream of MTL and was found to affect HIR in two independent GWAS studies [6,16].

Further studies may need to confirm or refute *MYO*’s correlation with HIR; however, results clearly establish the connection between *MYO* and male fertility/sterility, which has not been previously reported. Past studies have shown that there are many different myosin classes within plants, that all may have unique functions, especially considering many have specific localized expression [17]. Since *MYO* is expressed exclusively in the pollen, the function was expected to be related to male fertility; however, this is the first publication clearly establishing an association.

## 4. Materials and Methods

### 4.1. Identification of Gene of Interest

*GRMZM2G435294* or Myosin-11 (*MYO*) was considered as a candidate gene for *qhir8* because it is specifically expressed in anthers [18,19], co-expressed with *MTL* [8], and is located within the fine mapped region of *qhir8* [5]. *MYO* was amplified from the haploid inducer genotype RWS/RWK-76 [20] and cloned into the pGEM-T vector system (Promega, Fitchburg, WI, USA) for sequencing and alignment with the B73 reference genome. A 1059 bp region downstream of the start of exon 19 was sequenced and aligned to B73 RefGen_v3 [15] and analyzed for polymorphic regions (Table S1).

### 4.2. Production of Transgenic Materials

Silencing of *MYO* was accomplished by delivering transgenic RNA interference (RNAi) constructs. The target sequences were designed using pssRNAit software [21]. The best siRNA options were chosen based on the most efficient silencing effect, the fewest off-target hits, as well as their location within the gene. Two target regions were chosen within exon 16 (126 bp) and exon 18 (96 bp), and designated as *MYO*16 and *MYO*18, respectively (Table S2). Both constructs were expected to accomplish the same goal of silencing *MYO* and are therefore considered equivalent, if qPCR results show similar silencing levels.

Corresponding RNAi vectors were constructed for creating transgenic events. Primers containing appropriate flanking restriction sites (Table S2) were used to amplify a fragment of *MYO* to be inserted in sense (*Spe*I and *Sma*I) and anti-sense orientations (*Bst*Z17I and *Avr*II) within the pMCG1005 RNAi binary vector (obtained from the Plant Transformation Facility at ISU). This vector contains the first intron of maize alcohol dehydrogenase-1 (*AdhI*) upstream of the RNA hairpin-producing sequence, which is driven by the maize ubiquitin1 (*ubi1*) promotor. This vector also contains four copies of an enhanced CaMV 35S promoter that drives the bar gene. After these vectors were transformed via *Agrobacterium* into maize genotype HiII by the Plant Transformation Facility at ISU using the protocol described by Frame et al. [22], transgenic plants were identified based on glufosinate resistance. A total of 12 transgenic events were produced for each construct, but only 5 transgenic events (one for *MYO*16 and four for *MYO*18) are included in this study. These events were chosen for no other reason than early availability.

### 4.3. Development of Control Lines

Because *qhir1* is required for haploid induction [1,5,6] and *MYO* is evaluated as a candidate for *qhir8*, it was necessary to evaluate the effect of the transgene in the presence of *qhir1*. *qhir1*-containing materials were created by crossing RWS/RWK-76 [20] with B73 and then backcrossing with B73 until reaching the BC_2_ generation. Simple sequence repeat (SSR) markers were designed to flank *qhir1* (1.04_682414 and GSS_44), and public markers that flank *qhir8* (umc1040 and bnlg1272) were used to select both QTL regions (Table S3). In each backcross generation, individuals were selected for presence of *qhir1* and absence of *qhir8* (*^qhir1^*B73) by marker analyses. Additional control lines were developed by selection for presence of *qhir8* and absence of *qhir1* (*^qhir8^*B73) or presence of both QTL, *qhir1* and *qhir8* (*^qhir,1qhir8^*B73). Finally, BC_2_F_2_–derived lines were created to fix *qhir1* and/or *qhir8*, respectively.

### 4.4. Development of Transgenic Families

At the T0 stage, all events were crossed and then backcrossed to B73 (MSG 14786) [23] (*^MYO^*F_1_ and *^MYO^*BC_1_F_1_, respectively). Transgenic F_1_ offspring were selected by QualiPlate ELISA kit for the detection of PAT/bar (EnviroLogix, Portland, ME, USA), resulting in transgenic BC_1_ families segregating for hemizygous presence of the RNAi construct within the B73 genetic background.

**Figure 4:**
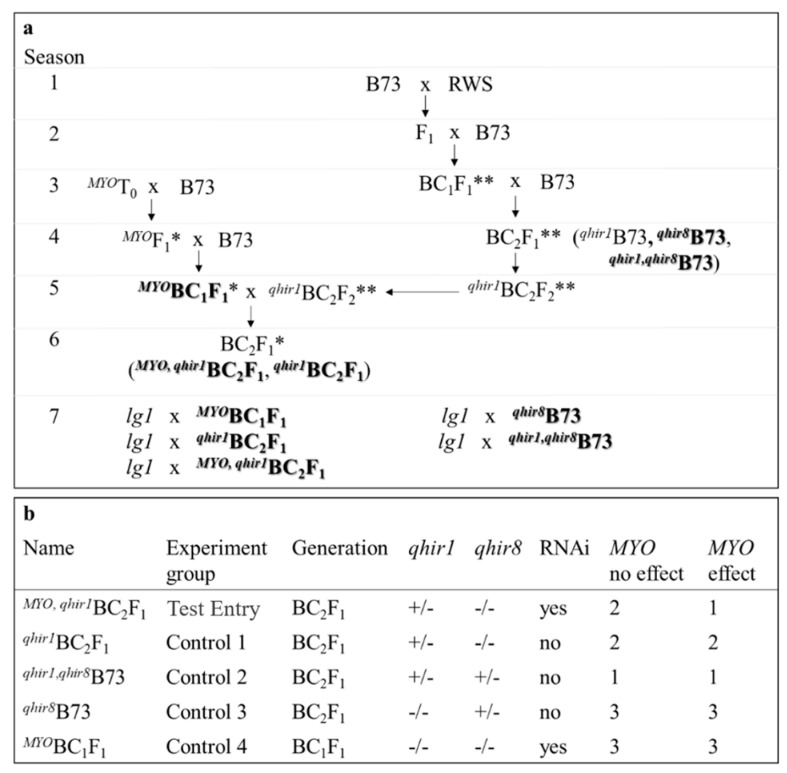
Schematic development of transgenic families and control groups (**a**). Bolded families are those developed for the test-cross with *lg1* (Season 7). Noted, are methods of selecting transgenic plants (ELISA PAT/bar; *) and those with desired QTL regions (marker selected for *qhir1* and/or *qhir8*; **). A summary of details regarding each group tested in HIR is also provided (**b**). Expected results are specified if *MYO* is not responsible for *qhir8* (*MYO* no effect) or if *MYO* is responsible for *qhir8* (*MYO* effect). Expected outcomes are numbered, with “1” noted for the highest HIR levels and “3” for the lowest HIR levels. Equal numbers imply that HIR values are comparable between the two experimental groups.

Each of the five transgenic BC_1_ families were crossed with *^qhir1^*B73. BC_1_ materials were screened and selected for the presence of the transgene before crossing with *^qhir1^*B73. Finally, the resulting BC_2_ materials were screened and classified into those containing (*^qhir1,MYO^*BC_2_F_1_) or not containing (*^qhir1^*BC_2_F_1_) the *MYO* construct. Therefore, *MYO* and *qhir1* are heterozygous in these lines. Both BC_1_ and BC_2_ offspring were screened for transgene presence with QualiPlate ELISA kit for the detection of PAT/bar (EnviroLogix, Portland, ME, USA) (Figure 4a). Controls included *^qhir8^*B73 (BC_2_F_1_), *^qhir1,qhir8^*B73 (BC_2_F_1_), and the *^MYO^*BC_1_F_1_ families. Prior to transplanting *^MYO^*BC_1_F_1_ families, the presence of the transgene was identified by spraying 0.5% glufosinate (190 L/ha).

### 4.5. Test Crossing and Evaluation of HIR

To analyze HIR, testcrosses were made between control inducers or potential inducers (male) and inbred line *lg1* (female) (205B, MGS 14013) in the summer of 2017 in Ames, IA. The line *lg1* contains B73 background and is homozygous recessive for ligule presence. Therefore, any haploids created by testcrosses can be identified by the lack of ligules.

### 4.6. Experimental Design and Statistical Analysis for HIR

This experiment was arranged in a modified incomplete block design including controls *^qhir8^*B73 and *^qhir1,qhir8^*B73, which were repeated in three biological replicates within each of three blocks to account for environmental variation between blocks. Transgenic families were included in two to four biological replicates within each block. Each replicate contained 10 plants, and when ≥5 plants were pollen shedding, pollen was bulked and used to randomly pollinate 3–6 *lg1* plants. The female parent carrying *lg1*, was planted in a large area beside the crossing block. Subsequently, an average of 375 offspring were grown out for each biological replicate of each genotype in the greenhouse for analysis of ligule presence at the two to three leaf stage. All replicate data from the same control or transgenic family were combined within each incomplete block.

Haploid induction rates were compared between families or to the reference value for the spontaneous HIR rate (0.1% or 1 haploid in every 1000 seedlings) [24,25] by proc LOGISTICS in SAS 9.4 (SAS Institute Inc.). The following model was used for variance analysis:

(n_h_/n_t_)_ij_ = µ + B_i_ + F_j_ + ε_ij_ (1)

where (n_h_/n_t_)_ijk_ represents the observation of the ijth experimental unit, or haploid induction rate (number of haploid plants (n_h_) divided by the total number of plants (n_t_)). Main effects are represented by B_i_ or the ith block, and F_j_ or the ith family. The error term is defined as ε_ij_. The significance test was performed using Walds chi-square test.

The mean HIR for each family was computed with proc GLIMMIX in SAS 9.4 (SAS Institute Inc.). The following model was used for variance analysis:

(n_h_/n_t_)_ijk_ = µ + B_i_ + F_j_ + C_k_ + FC_jk_ + ε_ijk_ (2)

where (n_h_/n_t_)_ijk_ represents the observation of the ijkth experimental unit, or haploid induction rate (number of haploid plants (n_h_) divided by the total number of plants (n_t_)). The main effects are represented by B_i_ or the ith block, F_j_ or the ith family, and C_k_ or the kth cross type (stage of backcrossing). The error term is defined as ε_ijk_.

To account for the potential contamination or accidental self-pollination of *lg1* donor plants, pollination sources that were three times the standard deviation for any cross combination were considered outliers and removed [26]. Data from one pollination for each of the two genotypes *MYO*16-1 and *MYO*18-2 (both *^qhir1^*BC_2_F_1_) were removed from the data set.

### 4.7. Male Fertility Analysis

Reciprocal crosses were made between four hemizygous F_1_ plants (*^MYO^*F_1_) of each transgenic event and randomly selected B73 plants in the summer of 2016. Offspring from these reciprocal crosses were grown out in trays of 126 seeds per cross (4 crosses × 126 seeds = 504 seedlings analyzed in total for each event). Seedlings were sprayed with 0.5% glufosinate (190 L/ha) at the 2–3 leaf stage, and after 3–4 days the effects were visible enough to count survival rates. Data were analyzed by proc MIXED in SAS 9.4. Accuracy of the test was evaluated by a chi-square test with an expected segregation ratio of 1:1 when B73 was the male parent. This was performed using proc LOGISTICS in SAS 9.4 (SAS Institute Inc., Cary, NC, USA).

### 4.8. Silencing Efficiency by Quantitative PCR

Expression analysis by qPCR was performed using the ΔΔC_T_ method [27]. For this method, the expression of transgenic events was compared to both a calibrator (wild type, i.e., Viking brand 60-01 by Albert Lea Seed) and a normalizer (housekeeping gene, i.e., *MEP*, Membrane protein PB1A10.07c) with the following equations:

ΔC_T_ = C_T,*MYO*_ − C_T,*MEP*_ (3)

ΔΔC_T_ = ΔC_T,trans_ − ΔC_T,Viking_ (4)

relative expression = 2^−ΔΔC^_T_ (5)

where C_T_ represents the designated sample’s cycle threshold, or the number of cycles required for the fluorescent signal to exceed the threshold level. Normalized expression is the expression after considering the house-keeping gene expression levels and is represented as ΔC_T_. Calibrated expression is the expression after considering expression levels for both the house-keeping gene and the reference genotype and is represented as ΔΔC_T_. Relative expression is the fold change in *MYO* expression between the genotype of interest and the reference genotype. Five transgenic (*^MYO^*BC_1_F_1_) families, segregating for the transgene, were grown in the greenhouse until the 2–3 leaf stage, sprayed with 0.5% glufosinate (190 L/ha) and surviving seedlings were transplanted to the field along with the Viking genotype. At approximately V10 stage, anthers were harvested for qPCR analysis. Three plants of appropriate size were harvested from Viking and from each transgenic family. Plant stalks were severed between nodes just above the ear shoots and stored in buckets of water, while being transported to the laboratory for anther dissection [28]. Since the upper anthers mature more quickly than the lower anthers, dissection was performed to harvest only the upper anthers for RNA extraction and qPCR analysis. Approximately 50–100 mg of dissected anthers, which ranged from 4–5 mm (binucleate microspore stage), were placed in a microfuge tube resting in liquid nitrogen. Samples were stored in −80 °C prior to grinding in liquid nitrogen for subsequent RNA extraction. RNA was purified using the RNeasy Plant Mini Kit (Qiagen, Germantown, MD, USA) with on-column DNase digestion by following manufacturer’s instructions. RNA concentrations were measured by a NanoDrop 2000c Spectrophotometer prior to Reverse transcription-PCR (RT-PCR) reactions. RT-PCR was performed with SuperScript^TM^ III First-Strand Synthesis System (Invitrogen by life technologies, Carlsbad, CA, USA) using oligo(dT)_20_ primers. From the final 20 µl reaction containing cDNA, 1 µl was used for further qPCR reactions (for a target cDNA concentration of 10 ng). Reactions for qPCR were performed using Power SYBR^TM^ Green PCR master mix (Applied Biosystems by life technologies, Foster City, CA, USA) and gene specific primers (Table S2) on a Mx3000P qPCR system. The primer pair chosen for *MYO* bridged an intronic region (81 bp) between exon 6 and 7 to further ensure the lack of DNA contamination during qPCR analysis. The primer pair utilized for *MEP*, the reference gene of choice, was published by Manoli et al. [29]. Gene-specific primers were optimized by conducting qPCR on a serial dilution (1 ng, 10 ng, 100 ng) with primer pairs for *MYO* and *MEP*. Primer optimization was performed by analyzing the linear trend of the difference between the expression of the *MYO* and *MEP* (ΔC_T_) as well as the analysis of standard curves for each individual primer pair. R^2^ values close to 1 indicate high amplification efficiency. The ΔC_T_ curve produced an R^2^ value of 0.95, whereas both individual primer pairs for *MYO* and *MEP* scored R^2^ values of 0.99. A three-step PCR program of 95 °C (15 s), 55 °C (30 s), and 68 °C (30 s) for 40 cycles was used for both primer pairs with a 25 uL reaction size. There were three biological and three technical replicates for each genotype by primer pair combination. Statistical comparisons were performed in R using the ‘pcr’ package.

## Figures and Tables

**Table 1:** qPCR results for different transgenic lines. Expression is normalized by house-keeping gene *MEP* and calibrated with Viking genotype.

Genotype	*p*-Values	Normalized Expression (ΔC_T_)	Calibrated Expression (ΔΔC_T_)	Relative Expression (Fold Change)
***MYO*** **16-1**	0.04 *	1.17	−2.35	5.09
***MYO*** **18-1**	0.05 *	1.80	−1.72	3.30
***MYO*** **18-2**	0.03 *	1.38	−2.14	4.40
***MYO*** **18-3**	0.03 *	1.34	−2.18	4.52
***MYO*** **18-4**	0.16	2.33	−1.19	2.29
**Viking**	-------	3.52	0.00	1.00

* indicates significance at the 0.05 level.

**Table 2:** Mean values and standard errors for HIR are reported along with the *p*-values, which indicate significance when compared to spontaneous HIR of 0.1% (* at the 0.05 level and ** at the 0.01 level).

Group	Cross ID	*p*-Value	HIR (%)	S.E.
**Controls**	Spontaneous		0.1000	
	*^qhir8^*B73	0.0007 **	0.2398	0.0734
	*^qhir1,qhir8^*B73	0.0016 **	1.157	0.1708
***MYO*16-1**	*^MYO^*BC_1_F_1_	0.0336 *	0.0829	0.0852
	*^qhir1^*BC_2_F_1_	0.0918	1.120	0.3356
	*^qhir1,MYO^*BC_2_F_1_	0.0291 *	1.250	0.3449
***MYO*18-1**	*^MYO^*BC_1_F_1_	0.1723	0.2333	0.1735
	*^qhir1^*BC_2_F_1_	0.5274	0.8517	0.3650
	*^qhir1,MYO^*BC_2_F_1_	0.0030 **	1.581	0.4294
***MYO*18-2**	*^MYO^*BC_1_F_1_	0.0958	0.1207	0.1245
	*^qhir1^*BC_2_F_1_	0.1120	1.216	0.4717
	*^qhir1,MYO^* BC_2_F_1_	0.0001 **	2.015	0.5555
***MYO*18-3**	*^MYO^*BC_1_F_1_	0.1893	0.2413	0.1794
	*^qhir1^* BC_2_F_1_	0.9402	0.6711	0.3063
	*^qhir1,MYO^* BC_2_F_1_	0.0429 *	1.254	0.3681

**Table 3:** Average offspring survival rate after herbicidal treatment, along with standard errors. *p*-values are also provided for transgenic events, female parent, and their interaction.

Male Parent	Survival Rate (%)	S.E.	Effect	*p*-Values
**B73**	47.45	1.16	**Event**	0.0001
***MYO*** **16-1**	0	1.77	**Female**	<0.0001
***MYO*** **18-1**	12.42	1.77	Event * Female	0.0001
***MYO*** **18-2**	20.11	2.97		
***MYO*** **18-3**	8.48	1.77		

* indicates interation between effects.

## Supplementary Materials

The following are available online at https://www.mdpi.com/2223-7747/9/6/773/s1, Figure S1: Correlations between relative expression (fold change) of *MYO* transgenic events and (**a**) survival rates after spraying a herbicidal treatment for transgene selection (data for B73 × *^MYO^*F_1_ crosses), or (**b**) haploid induction rates (% haploids found for *^qhir1,MYO^*BC_2_F_1_), Table S1: Protein alignment of B73 RefGen_v3 [15] with RWS. Sequenced region is shaded in grey, and remaining gaps were assumed to match reference sequence. Highlighted sequences indicate polymorphisms, Table S2: Sequence information for RNAi targets and primers, Table S3: Primers used for SSR marker analysis and the presence or absence of QTL regions. Relevant information provided here is based on B73 RefGen_v4 [30].
