# Supplementary material for: MYO, a Candidate Gene for Haploid Induction in Maize Causes Male Sterility
Source: Plants (Basel). 2020 Jun 19;9(6):773. doi: 10.3390/plants9060773 (PMC7355785; doi:10.3390/plants9060773)
Supplement: Supplementary file 1 [file plants-09-00773-s001.pdf]

SUPPLEMENTARY INFORMATION

Table 1. Protein alignment of B73 RefGen_v3 [15] with RWS. Sequenced region is shaded in grey, and remaining gaps were assumed to match reference sequence. Highlighted sequences indicate polymorphisms.

```
B73   MGTKVNIIVGSHVWAEDPDTCWVDGEVVKINGEEAEIQATNGKKIVANLSKLYPKDMEAA  60
RWS   MGTKVNIIVGSHVWAEDPDTCWVDGEVVKINGEEAEIQATNGKKIVANLSKLYPKDMEAA  60
      ************************************************************

B73   AGGVDDMTKLSYLHEPGVLQNLAIRYELNEIYTYTGNILIAVNPFQRLPHLYDPHMMHQY  120
RWS   AGGVDDMTKLSYLHEPGVLQNLAIRYELNEIYTYTGNILIAVNPFQRLPHLYDPHMMHQY  120
      ************************************************************

B73   KGAPFGELSPHVFAVADVAYRAMVNENKSNAILVSGESGAGKTETTKMLMRYLAYLGGRA  180
RWS   KGAPFGELSPHVFAVADVAYRAMVNENKSNAILVSGESGAGKTETTKMLMRYLAYLGGRA  180
      ************************************************************

B73   ATEGRTVEQQVLESNPVLEAFGNAKTVRNNNSSRFGKFVEIQFDKHGRISGAAIRTYLLE  240
RWS   ATEGRTVEQQVLESNPVLEAFGNAKTVRNNNSSRFGKFVEIQFDKHGRISGAAIRTYLLE  240
      ************************************************************

B73   RSRVCQVSDPERNYHCFYLLCAAPQEDVDKYKLGNPKTFHYLNQSNCYELVGVSDAHEYL  300
RWS   RSRVCQVSDPERNYHCFYLLCAAPQEDVDKYKLGNPKTFHYLNQSNCYELVGVSDAHEYL  300
      ************************************************************

B73   ATRRAMDIVGISTQEQDAIFRVVAAILHIGNIEFSKGKEADSSVLKDEKSKFHLETTAEL  360
RWS   ATRRAMDIVGISTQEQDAIFRVVAAILHIGNIEFSKGKEADSSVLKDEKSKFHLETTAEL  360
      ************************************************************

B73   LMCNPGALEDALCKRVMVTPEEVIKRSLDPYNATISRDGLAKTIYSRLFDWLVDKINSSI  420
RWS   LMCNPGALEDALCKRVMVTPEEVIKRSLDPYNATISRDGLAKTIYSRLFDWLVDKINSSI  420
      ************************************************************

B73   GQDASSKCLIGVLDIYGFESFKANSFEQFCINYTNEKLQQHFNQHVFKMEQEEYTKEQID  480
RWS   GQDASSKCLIGVLDIYGFESFKANSFEQFCINYTNEKLQQHFNQHVFKMEQEEYTKEQID  480
      ************************************************************

B73   WSYIEFVDNQDVLDLIEKKPGGVIALLDEACMFPKSTHETFAQKLYQTFQKHKRFVKPKL  540
RWS   WSYIEFVDNQDVLDLIEKKPGGVIALLDEACMFPKSTHETFAQKLYQTFQKHKRFVKPKL  540
      ************************************************************

B73   SRTDFTICHYAGEVLYQSDQFLDKNKDYVVAEHQELLSASKCSFISGLFPPPPEETSKSS  600
RWS   SRTDFTICHYAGEVLYQSDQFLDKNKDYVVAEHQELLSASKCSFISGLFPPPPEETSKSS  600
      ************************************************************

B73   KFSSIGARFKQQLQALMDTLNSTEPHYIRCVKPNNVLKPAIFENVNVMQQLRCGGVLEAI  660
RWS   KFSSIGARFKQQLQALMDTLNSTEPHYIRCVKPNNVLKPAIFENVNVMQQLRCGGVLEAI  660
```

```
         ************************************************************

B73     RISCAGYPTRRTFYEFLHRFGILAPEALEGNSDEKAACKRILEKKGLLGFQIGKTKVFLR    720
RWS     RISCAGYPTRRTFYEFLHRFGILAPEALEGNSDEKAACKRILEKKGLLGFQIGKTKVFLR    720
         ************************************************************

B73     AGQMAELDARRTEVLSAAAKTIQGKMRTHIMRKKFLSLRKASVCVQAIWRG-RLACKLYD    779
RWS     AGQMAELDARRTEVLSAAAKTIQGKMRTHIMRKKFLSLRKASVCVQAIWRGGRLACKLYD    780
         *************************************************** ********

B73     NMRREAAAIKVQKNQRRHQARRSYKLRYASVLVVQTALRAMAARNEFRFKKQSTGAVTIQ    839
RWS     NMRREAAAIKVQKNQRRHQARRSYKLRYASVLVVQTALRAMAARNEFRFKKQSTGAVTIQ    840
         ************************************************************

B73     ARYRCYRAHKYHKKLKCAAIVAQCRWRGRIARKELKKLKMEARETGALKEAKDKLEKKVE    899
RWS     ARYRCYRAHKYHKKLKCAAIVAQCRWRGRIERRTNSKRKGR------------------    881
         ****************************** *:  .* * .

B73     ELTWRVQLEKRLRTDLEEAK---AQEVSKLQNSMEALQAKLDETNTKLAKEREAAKTIEE    956
RWS     -TPWRAVR-KRLRDRPGRSKSSRGVETAELYGSITG------------------------    915
           **.  ****   .:*   . *.:.* .*:. .

B73     APPVVQETQVLVQDTEKIDSLTAEVQDLKTSLQSEKERAGDLEKKHSEEQQANEEKQKKL    1016
RWS     ------------------------------------------------------------    915

B73     DETEIKMRQFQDYLRRLEEKLANVESENKVLRQQAVSMAPSKILSGRSKSNLQRNSENVQ    1076
RWS     ------------------------------------------------------------    915

B73     VSSNDPKTAPESNSTSSPKKEYDIDDKPQKSLNEKQQENQDLLIRCIAQHLGYAGNRPVA    1136
RWS     ------------------------------------------------------------    915

B73     ACIIYKCLLHWRSFEVERTSVFDRIIQTIGHAIETQDNNEVLAYWLSNASTLLLLLQRTL    1196
RWS     ------------------------------------------------------------    915

B73     KASGSTGMAPQRRRSSSATLFGRMTQSFRGAPQGVNLSLINGSMVTGVETLRQVEAKYPA    1256
RWS     ------------------------------------------------------------    915

B73     LLFKQQLTAYVEKIYGMIRDNLKKEISPLLGLCIQAPRTSRASLMKGSSRSNTNTAAQQA    1316
RWS     ------------------------------------------------------------    915

B73     LIAHWQGIVKSLGNFLNILKVNNVPPFLVRKVFTQIFSFINVQLFNSLLLRRECCSFSNG    1376
RWS     ------------------------------------------------------------    915

B73     EYVKAGLAELEHWCYRATDEYAGSAWDELKHIRQAIGFLVIHQKPKKTLDEISHDLCPVL    1436
RWS     ------------------------------------------------------------    915
```

B73 SIQQLYRISTMYWDDKYGTHSVSPEVISNMRVLMTEDSNNPISNSFLLDDDSSIPFSVDD 1496
RWS -- 915

B73 ISKSMQQIDISDIEPPPLIRENSGFVFLLPPPE 1529
RWS --------------------------------- 915

Table S2. Sequence information for RNAi targets and primers

Name	Sequence	Product Size (nt)	Purpose
**MEP_F**	TGTACTCGGCAATGCTCTTG	203	qPCR primer
**MEP_R**	TTTGATGCTCCAGGCTTACC		
**MYO_F**	TGACCCAGAGCGCAATTAC	145	qPCR primer
**MYO_R**	ATCACTTACACCGACCAACTC		
**MYO18_F**	TTTCCTAGGACTAGTGTGTGCTTGAGGCCATCAG	96	RNAi primer
**MYO18_R**	TTTGTATACCCCGGGCCTCAGGCGCAAGTATTCC		
**MYO16_F**	TTTCCTAGGACTAGTCAAAGACTACGTTGTGGCGG	126	RNAi primer
**MYO16_R**	TTTGTATACCCCGGGGCTCCAATGGAAGAGAACTTGG		
**MYO18 Target**	GTGTGCTTGAGGCCATCAGGATCAGTTGTGCCGGGTACCCGACGCGTCGCACATTCTACGAGTTTCTGCATCGTTTCGGAATACTTGCGCCTGAGG		
**MYO16 Target**	CAAAGACTACGTTGTGGCGGAGCACCAGGAACTGCTGAGCGCTTCTAAATGCTCGTTTATCTCCGGATTGTTTCCGCCTCCGCC		

AGAGGAGACGTCCAAATCGTCCAAGTT
CTCTTCCATTGGAGC

Table 3. Primers used for SSR marker analysis and the presence or absence of QTL regions. Relevant information provided here is based on B73 RefGen_v4 [30].

Marker Name	QTL	Bin	Product Length (bp)	Anneal Temp (ºC)	Sequence	Starting Coordinates	Relevant Gene
1.04_682414	*qhir1*	1.04	150-200	57	Fwd: TTGTTCTTGCATCCATCCAG Rev: CATGCACTTGCCGTTGTACT	69429443	within *GRMZM2G471240*
GSS_44	*qhir1*	1.04	150-200	57	Fwd: AATGTGGCATGTACGAGGTG Rev: AGGGGTTAGGCGGATCAAT	69445286	downstream *GRMZM2G471240*
umc1040	*qhir8*	9.01	100-200	57	Fwd: CATTCACTCTCTTGCCAACTTGA Rev:AGTAAGAGTGGGATATTCTGGGAGTT	4446555	overlapping *GRMZM2G124276*
bnlg1272	*qhir8*	9.01	250	57	Fwd: ACCGAAGATGAGGTGTGACA Rev: TCAGTGCAAGGGCAATTTAG	85302	downstream *GRMZM2G310569*

Figure 1. Correlations between relative expression (fold change) of *MYO* transgenic events and a) survival rates after spraying a herbicidal treatment for transgene selection (data for B73 x $^{MYO}F_1$ crosses), or b) haploid induction rates (% haploids found for $^{qhir1,MYO}BC_2F_1$). .
